# A Practical Assessment of the Postoperative Management in Lung Cancer Surgery

**DOI:** 10.3390/jpm13020358

**Published:** 2023-02-18

**Authors:** Luca Bertolaccini, Shehab Mohamed, Clarissa Uslenghi, Matteo Chiari, Lara Girelli, Giorgio Lo Iacono, Lorenzo Spaggiari

**Affiliations:** 1 Department of Thoracic Surgery, IEO, European Institute of Oncology IRCCS, Via Ripamonti 435, 20141 Milan, Italy; 2 Department of Oncology and Hemato-Oncology, University of Milan, 20122 Milan, Italy

**Keywords:** lung cancer, postoperative management, thoracic surgery

## Abstract

Postoperative management after major and minor thoracic surgeries is crucial for patient recovery and can be challenging. Major thoracic surgeries, such as extensive pulmonary resections, especially in patients with poor health status, may require intensive surveillance, particularly during the first 24–72 h after surgery. Moreover, thanks to the demographic development and medical progress in perioperative medicine, more patients with comorbidities undergoing thoracic procedures require proper management in the postoperative period to improve prognosis and decrease hospital stay. Here, we summarize the main thoracic postoperative complications in order to clarify how to prevent them through a series of standardized procedures.

## 1. Introduction

Postoperative management after major and minor thoracic surgeries is crucial for patient recovery and can be challenging. Major thoracic surgeries, such as extensive pulmonary resections, especially in patients with poor health status, may require intensive surveillance, particularly during the first 24–72 h after surgery. Moreover, thanks to demographic development and medical progress in perioperative medicine, more patients with comorbidities undergoing thoracic procedures require proper management in the postoperative period to improve prognosis and decrease hospital stay.

Several hospital professionals and thoracic surgeons are involved in reaching proper postoperative care, such as anesthetists, cardiologists, pneumologists, physiotherapists, specialized nurses, logopedics, and other specialists. A good collaboration between these figures is essential to improve patient outcomes and manage potential postoperative complications in the best way possible.

Several points must be emphasized and considered for proper management: pulmonary hygiene and physical therapy, fluid balance, chest tube management, and pain control. These aspects are essential to prevent medical complications such as respiratory failure and infections, atelectasis, arrhythmias, and venous thromboembolism. Furthermore, even though these complications can present singularly, two or more can coincide in the most fragile patients. In this last scenario, the cooperation between all the hospital figures becomes even more critical for stabilizing the patients. 

This opinion paper will provide a summary of the most common thoracic postoperative problems and explain how to prevent them using a series of standardized protocols for thoracic surgeons and other specialists. In this study, an evidence-based multimodal approach (enhanced recovery after surgery—ERAS) was used to evaluate all these key postoperative measures to reduce the stress response to surgery, complications, and hospital length of stay following surgery [1]. Several studies have demonstrated that the ERAS procedure can effectively prevent postoperative problems, enhance the quality of life before and after surgery, and shorten hospital stays [2,3].

## 2. Pulmonary Infections and Physical Therapy

Pneumonia is the most potentially severe early postoperative complication, particularly for individuals undergoing open surgery. Atelectasis, which results in secretion retention and localized hypoventilation, is a significant risk factor for lung infections. Chest wall pain may decrease pulmonary ventilation during open thoracic procedures, encouraging atelectasis. Preventing pulmonary infections and atelectasis is possible through preoperative preparation and postoperative treatment.

In the preoperative period, smoking is related to an increased risk of postoperative morbidity and mortality; therefore, smoking should be discontinued at least four weeks before surgery. Prehabilitation is a further consideration that must be made. A preoperative exercise rehabilitation program should be considered to shorten hospital stays, particularly for patients with impaired lung function or exercise ability before surgery. Due to the small number of studies and their heterogeneity, no recommendations can be made about its mode, frequency, and duration. Unfortunately, preoperative antibiotic prophylaxis used to reduce surgical site infection and taken no more than 60 min before skin incision had little effect on postoperative pneumonia, according to data on preoperative drugs [4]. Furthermore, there is little indication that adding preoperative short-term bronchodilators alters surgical results.

Several approaches for preventing atelectasis in the postoperative phase have been documented, and physiotherapy plays a crucial role in the rehabilitation of thoracic surgery patients. Continuous positive airway pressure (CPAP), incentive spirometry, and airway-clearing procedures such as coughing and deep breathing are the most prevalent strategies that have been found to reduce the incidence of atelectasis and avoid bacterial pneumonia after surgery. These procedures permit patients to maintain deep, sustained respiration, which is believed to recruit collapsed alveoli and recover preoperative pulmonary function. Additionally, nasal cannulae should be used to give supplemental oxygen as needed during the postoperative period to maintain oxygen saturation greater than 92%. However, it should be kept in mind that excessive oxygen administration may have negative consequences. Increased alveolar oxygen tension promotes atelectasis due to the fast absorption of oxygen.

There is scant evidence for incentive spirometry in the scientific literature, and different studies doubt its clinical utility for thoracic surgery patients. Patients with a greater risk of acquiring postoperative pneumonia, such as those with chronic obstructive pulmonary disease (COPD), could potentially get a benefit; nevertheless, this aspect requires additional investigation. It appears that spirometry, when utilized successfully, can serve as a powerful incentive for patients to continue completing breathing exercises and contribute to minimizing the incidence of postoperative pulmonary problems by reducing atelectasis. However, proving this theory in clinical studies remains difficult. With sufficient preoperative training and use as a component of multidisciplinary therapy, it is possible that incentive spirometry could be a helpful intervention with benefits extending beyond the hospital stay.

In addition, respiratory therapists play a crucial role in reducing postoperative pulmonary problems and are regarded as standard postoperative treatment, particularly for patients undergoing open thoracic surgery. By applying CPAP early, the incidence of atelectasis and the requirement for intubation and mechanical ventilation can be decreased in patients with hypoxemia. The latter raised functional residual capacity more rapidly than the other techniques, but Stock and colleagues describe it as intolerable [5]. Compliance with these procedures is essential for optimal outcomes, particularly for patients with coexisting conditions. Consequently, selecting the best approach for preventing pulmonary problems is crucial.

Before and after surgery, patients should be encouraged to complete home exercises, incentive spirometry, and particular inspiratory muscle training [6]. This remains the most effective method for preventing atelectasis, and physical treatment, including early ambulation, helps to prevent these problems after surgery.

## 3. Thromboembolism

Venous thromboembolism is another frequent surgical consequence. Early mobilization is a prophylaxis that includes mechanical measures (like elastic compression stockings and intermittent pneumatic compression devices) and pharmaceutical interventions (like low-dose unfractionated or fractionated subcutaneous heparin). The risk of getting deep venous thrombosis is increased for all individuals receiving major surgical interventions. In this instance, elastic compression stockings worn before surgery and early mobilization, typically on the same day or the day after, can reduce the risk of deep venous thrombosis and pulmonary embolism. Mechanical and pharmacological prophylaxis is the best standard of care for preventing deep venous thrombosis in high-risk individuals and can be used in combination. In low-risk patients, the American Society of Clinical Oncology (ASCO) recommends that unfractionated subcutaneous heparin in modest doses (5000 UI) be administered 2 h before surgery and continued every 12 h for at least 7 to 10 days after surgery. Patients who are taking anticoagulants prior to surgery require special consideration. In general, heparin can be administered to these patients 6 to 12 h after surgery, depending on intraoperative blood loss and the need for anticoagulation. Patients with low-risk conditions, such as atrial fibrillation without a past stroke or cardiomyopathy without atrial fibrillation, can begin anticoagulation following hospital discharge. Before discharge, moderate-risk patients (such as those with a mechanical aortic valve) must resume anticoagulant treatment. Patients at high risk (such as those with a mechanical mitral valve or atrial fibrillation with a history of stroke) must be anticoagulated with intravenous heparin as soon as feasible following surgery.

Implantation of inferior vena cava filters may be considered in high-risk individuals when pharmacologic prophylaxis is impractical and in patients who bleed when taking anticoagulants. The latter is often reserved for individuals who have just been diagnosed with deep vein thrombosis or pulmonary embolism [7].

## 4. Cardiac Arrhythmias

In addition, cardiac arrhythmias, particularly atrial fibrillation, are postoperative problems that a thoracic surgeon should anticipate. Atrial fibrillation can result in palpitations, tiredness, angina or infarction, hypotension, congestive heart failure, stroke, and more extended hospitalization, hence increasing the expenditures associated with hospital stays. Because this complication is more prevalent in cardiac surgery patients, the European Association for Cardio-Thoracic Surgery (EACTS) treatment guidelines might be revised for thoracic surgery patients [8]. Electrocardiography should be used to rule out myocardial ischemia as part of the standard of care.

As indicated in established procedures for Advanced Cardiac Life Support (ACLS), the treatment of atrial fibrillation focuses on the resuscitation of hemodynamically unstable patients. After establishing a diagnosis of atrial fibrillation, the objective is to evaluate the patient’s hemodynamic stability. In the event of syncope with a systolic blood pressure of less than 80 mmHg, the options are either electrical cardioversion or immediate chemical conversion. If the patient is hemodynamically stable, the physician should, if possible, use antiarrhythmics to modulate the ventricular rate to allow for more excellent ventricular filling and an appropriate ejection fraction. Digoxin, metoprolol, diltiazem, and verapamil have been used to regulate ventricular rate in patients with atrial fibrillation since they all inhibit atrioventricular nodal conduction. Unfortunately, patients undergoing thoracic surgery may experience bronchospasm. As a result of their detrimental effect on the bronchi, beta-adrenergic blockers are contraindicated. Amiodarone is one of the most commonly used medicines for cardioversion, primarily due to its relative safety in patients with decreased ventricular function. Thankfully, postoperative atrial fibrillation is brief and self-limiting; thus, amiodarone exposure is limited. When used chronically, this can prevent harmful effects such as lung fibrosis and thyroid toxicity.

Patients with numerous atrial arrhythmias who convert to sinus rhythm should continue taking their antiarrhythmic medication for at least 30 days after surgery. Patients can spontaneously convert to sinus rhythm in some instances. If atrial fibrillation persists for more than 30 days following surgery, outpatient electrical cardioversion should be offered if the patient is anticoagulated therapeutically.

## 5. Pain Management

Pain after thoracic surgery is frequently severe and usually caused by thoracotomy, injury to the intercostal nerves, fractures or dislocation of the ribs, and chest tube placement; therefore, adequate pain control is essential for early mobilization, deep breathing, and coughing of the patient, as well as for preventing undesirable complications such as respiratory failure, hypoxemia, arrhythmias, ischemia, and the development of post-thoracotomy pain syndrome.

There are numerous methods for managing pain, and a multimodal approach is necessary to keep the patient comfortable. They include preoperative epidural catheter implantation, regional anesthesia, and intravenous patient-controlled analgesia. In order to prevent problems such as atelectasis and deep vein thrombosis, the chosen form of pain treatment should stimulate participation in mobility and pulmonary rehabilitation.

Utilizing an epidural catheter in the operating room to provide preoperative analgesia and adequate pain control immediately after the operation with a combination of local analgesics and opiates is the standard of care for controlling pain after thoracic surgeries, particularly open surgeries with thoracotomies. Until chest tubes are withdrawn, epidural analgesia or a paravertebral thoracic catheter is typically maintained. Various consequences of epidural analgesia, including epidural hematoma, respiratory depression, and hypotension, have been recorded. Due to the development of video-assisted thoracic procedures and new analgesic approaches (such as paravertebral catheters), epidural analgesia has become less necessary in recent years. Paravertebral catheters may offer a safety advantage over epidural catheters for pain control in patients requiring anticoagulation due to the risk of epidural hematoma. Catheters placed in the paravertebral thoracic region permit the injection of local anesthetics, such as lidocaine, and can cover two dermatomes above and below the incision.

For patients undergoing minor surgical procedures, video-assisted thoracic surgery (VATS), or patients with coagulopathy, intercostal blocking with longer-acting bupivacaine that can be combined with adrenalin to delay absorption is an additional local anesthetic option to consider.

On the other hand, intravenous infusion of opioid medicines or oral narcotic compounds could reduce pain, particularly in patients having open thoracic surgeries or with sensitivities to acetaminophen or nonsteroidal anti-inflammatory treatments.

Different administration tactics for intravenous narcotics include hourly infusion, patient-controlled analgesia (PCA), and continuous infusion of medicines such as tramadol. Each of these methods can provide a minimally acceptable level of pain alleviation.

Oral opioids, such as oxycodone and codeine, are frequently used to treat withdrawal from epidural or intravenous treatment. In addition, these medications are typically necessary for a few days to a few weeks after hospital discharge, depending on patient factors such as adverse effects, the type of surgical technique, and the efficacy of symptom control.

Lastly, it is essential to remember that opioids can induce undesirable effects such as excessive drowsiness, confusion (especially in senior patients), respiratory depression, addiction, urine retention, and constipation difficulties that can lead to obstipation, perforation, and even death. Given that constipation is a common reason for readmission after thoracic surgeries, several preventative measures are available, such as stool softeners, early mobilization, and a progressive laxative regimen to protect bowel function soon after surgery.

## 6. Fluid Management

Patients undergoing lung resection procedures, particularly pneumonectomy, might develop interstitial and alveolar edema, which limits oxygen diffusion capacity. Restrictive fluid management is essential during and after thoracic surgery operations until IV fluids are replaced with oral fluids and nutrition. In patients undergoing pulmonary resections, excessive fluid replacement should not be administered. Lung parenchyma is deflated during thoracic procedures, which may compromise pulmonary lymphatic drainage and increase extravascular lung water due to disruption of the alveolar–capillary membrane. Excessive fluid administration can cause pulmonary edema, decreased alveolar gas permeability, atelectasis, and hypoxia. In this view, a proper assessment of fluid balance is essential, including the amount of fluid administered with drugs.

During surgery, particular concern must be given to patients with renal failure and considerable blood loss or fluid resuscitation. Close monitoring of urine output, serum electrolytes, calcium and magnesium, blood urea nitrogen, and creatinine is required in these instances. In addition, nephrotoxic medications such as non-steroidal anti-inflammatory drugs (NSAID) or angiotensin-converting enzyme inhibitors must be reevaluated or discontinued to reduce the risk of kidney damage.

Radiological examinations and laboratory testing could be used routinely on all patients to detect potential consequences of fluid imbalance and its treatment. Radiographs of the chest are essential for identifying pulmonary edema and pleural effusion. Low serum electrolyte levels may be associated with severe arrhythmias; therefore, periodic monitoring is required, particularly for diuretic-treated patients.

## 7. Chest Tube Management

The drainage tubes must be evaluated daily for functionality, air leakage, and drainage. After thoracic surgery, most surgeons insert one or two chest tubes to empty the pleural space and encourage pulmonary re-expansion. These tubes are connected to drainage systems that can give suction if the surgeon desires. The use of suction remains controversial in the literature; conversion to a standard water seal without suction is required only when considerable air leaks (>1500 mL/min) are detected. Patients having pneumonectomy are the only exception to aggressive suction at any moment. Regarding secretions, the removal of chest tubes appears to be well tolerated, even in the presence of a daily secretion greater than 450 mL [9,10]. Once the lung has fully expanded, and the air leak has ceased, chest tubes are also removed.

Due to the likelihood of malfunction and clogging, care must be taken with the drainage system. Generally, water seal and respiratory movements are synchronized in the drainage system; a blockage will hinder the water column and the respiratory motions. If this occurs, the external tube system must be examined for clamping or obstructions caused by purulent material, blood, or fibrin in the lumen of the chest tube. In this instance, the clog can be removed with irrigation, or the drainage tube can be replaced totally.

Air leaks, defined as leaking from the lung parenchyma into the pleural space, continue to be one of the most prevalent consequences of thoracic surgery. The bubbling of the water column during respiration suggests the presence of an air leak. Air leaks from the drainage system must be ruled out first, which can be accomplished by sequentially clamping the system. Once the clamp is positioned distal to the leak, the bubbling ceases.

Managing prolonged air leaks is possible through a variety of methods. Chest tube suction is modest or unnecessary if complete pulmonary re-expansion cannot be achieved. Fixing an air leak can take a while, so patience is required. In these instances, Heimlich valves allow patients to be discharged with minimal air leakage and uncomplicated access to hospital care. If this strategy is not feasible, reoperation may be undertaken to repair air leaks and prevent nosocomial infections.

Under the guidance of a computed tomography (CT) scan, a second chest drain placement can be performed in the remaining pockets where intrapleural air prevents the lung from fully re-expanding. Once all remaining air pockets are absorbed, and pleurodesis is accomplished, the air leak may cease immediately. Other pleurodesis techniques include the instillation of chemicals that produce pleural inflammation, such as doxycycline, the patient’s blood, or talc. Typically, a chest radiograph is used to assess pulmonary inflation. If the air leak persists for more than ten days, the suction can be lowered gradually, and if the lung stays inflated, the chest tube can be converted to a water seal and removed following a 24–48 h chest tube clamp. It is assumed that there is evidence of a stable localized pneumothorax in conjunction with an inflated lung over several days. In such a circumstance, the patient may be discharged, and the remaining bed space should gradually disappear over the following weeks.

Chest tubes are one of the leading sources of postoperative discomfort caused by intercostal nerve injury, and their removal appears to hasten recovery. Upon removal of the chest tube, pulmonary ventilation, mobility, and discomfort may improve. After the chest tube is removed, a chest radiograph is typically performed to determine if pleural apposition has been achieved.

## 8. Conclusions

A proper preoperative assessment of the patient for risk factors for surgery is crucial to achieving an excellent postoperative course. Generally, the quality of life after surgeries must be considered the most crucial outcome measure, and successful postoperative care should facilitate the patient’s return to full function. Different figures, such as surgeons, anesthesiologists, and nurses, must coordinate during and even after thoracic surgery, whether in an intensive care unit or a general ward bed. This experienced team must work in all these steps efficiently and well. Antibiotics for perioperative skin infections, anticoagulation strategies for deep venous thrombosis, and beta-adrenergic blockers for cardiac arrhythmias are examples of specific preventative measures that have been described following established principles and are beneficial. Patients discharged from the hospital may receive care suited to local practice, while keeping in mind the general objectives of early mobilization, excellent pain control, and speedy return to daily life.

## Data Availability

Not applicable.
